# Supplementary material for: Antagonistic control of Caenorhabditis elegans germline stem cell proliferation and differentiation by PUF proteins FBF-1 and FBF-2
Source: eLife. 2020 Aug 17;9:e52788. doi: 10.7554/eLife.52788 (PMC7467723; doi:10.7554/eLife.52788)
Supplement: Supplementary file 1. [file elife-52788-supp1.docx]

**Supplementary Table 1**

Nematode strains used in this study

| Genotype | Transgene description | Strain | Reference |
| --- | --- | --- | --- |
| **Mutant or CRISPR-Edited Strains; no transgene** | | | |
| *fbf-1(ok91) II* | - | JK3022 | (Crittenden et al., 2002) |
| *fbf-2(q738) II* | - | JK3101 | (Lamont et al., 2004) |
| *glp-1(ar202) III* | - | GC833 | (Pepper et al., 2003) |
| *fbf-1(ok91) II; glp-1(ar202) III* | - | UMT321 | this study |
| *fbf-2(q738)/mIn1[mIs14 dpy-10(e128)] II; glp-1(ar202) III* | - | UMT336 | this study |
| *pgl-1::gfp(ax3122) IV* | - | JH3269 | (Putnam et al., 2019) |
| *fbf-1(ok91) II; pgl-1::gfp(ax3122) IV* | - | UMT434 | this study |
| *fbf-2(q738) II; pgl-1::gfp(ax3122) IV* | - | UMT433 | this study |
|  | | | |
| **Transgenes; GFP::H2B::3’UTR** | | | |
| *rrf-1(pk1417) I; axIs1922[pCM1.252]* | *pie-1* prom::GFP::H2B::*htp-2* 3’UTR + *unc-119(+)* | UMT403 | this study |
| *rrf-1(pk1417)/hT2 [bli-4(e937) let-?(q782) qIs48] I; fbf-1(ok91) II; axIs1922[pCM1.252]* | *pie-1* prom::GFP::H2B::*htp-2* 3’UTR + *unc-119(+)* | UMT408 | this study |
| *rrf-1(pk1417) I; fbf-2(q738) II; axIs1922[pCM1.252]* | *pie-1* prom::GFP::H2B::*htp-2* 3’UTR + *unc-119(+)* | UMT393 | this study |
|  | | | |
| **Transgenes; ORF+3’UTR** | | | |
| *fbf-2(q738) II; mntSi33 (pXW6.29; 3xFLAG::CYB-2.1) unc-119(ed3) III* | *gld-1* prom::3xFLAG::CYB*-*2.1::*cyb-2.1 3’UTR* + *unc-119(+)* | UMT446 | this study |
| *fbf-2(q738) II; mntSi29 (pXW6.30; 3xFLAG::CYB-2.1fbm) unc-119(ed3) III* | *gld-1* prom::3xFLAG::CYB*-*2.1::*cyb-2.1 3’UTR(fbm) + unc-119(+)* | UMT406 | this study |
| *fbf-2(q738) II; mntSi33 (pXW6.29; 3xFLAG::CYB-2.1); pgl-1::gfp(ax3122) IV* | *gld-1* prom::3xFLAG::CYB*-*2.1::*cyb-2.1 3’UTR* + *unc-119(+)* | UMT448 | this study |
| *fbf-2(q738) II; mntSi29 (pXW6.30; 3xFLAG::CYB-2.1fbm) unc-119(ed3) III; pgl-1::gfp(ax3122) IV* | *gld-1* prom::3xFLAG::CYB*-*2.1::*cyb-2.1 3’UTR(fbm) + unc-119(+)* | UMT444 | this study |
| *mntSi23 (pXW6.24; 3xFLAG::CCF-1) II; unc-119(ed3) III* | *gld-1* prom::3xFLAG::CCF-1::*ccf-1 3’UTR* + *unc-119(+)* | UMT360 | this study |
| *mntSi23 (pXW6.24; 3xFLAG::CCF-1) II; mntSi28 (pXW6.27; patcGFP::FBF-1) unc-119(ed3) III* | *gld-1* prom::3xFLAG::CCF-1::*ccf-1 3’UTR* + *unc-119(+); gld-1* prom::patcGFP::FBF-1::*fbf-1 3’UTR* + *unc-119(+)* | UMT413 | this study |
| *mntSi23 (pXW6.24; 3xFLAG::CCF-1) II; mntSi27 (pXW6.26; patcGFP::FBF-2) unc-119(ed3) III* | *gld-1* prom::3xFLAG::CCF-1::*ccf-1 3’UTR* + *unc-119(+); gld-1* prom::patcGFP::FBF-2::*fbf-2 3’UTR* + *unc-119(+)* | UMT385 | this study |
| *mntSi23 (pXW6.24; 3xFLAG::CCF-1) II; mntSi32 (pXW6.32; patcGFP::FBF-2vrm) unc-119(ed3) III* | *gld-1* prom::3xFLAG::CCF-1::*ccf-1 3’UTR* + *unc-119(+); gld-1* prom::patcGFP::FBF-2(vrm)::*fbf-2 3’UTR* + *unc-119(+)* | UMT442 | this study |
| *mntSi23 (pXW6.24; 3xFLAG::CCF-1) II; mntSi21 (pXW6.22; patcGFP) unc-119(ed3) III* | *gld-1* prom::3xFLAG::CCF-1::*ccf-1 3’UTR* + *unc-119(+); gld-1* prom::patcGFP::*fbf-1 3’UTR* + *unc-119(+)* | UMT416 | this study |
| *fbf-1(ok91) fbf-2(q704) II; mntSi28 (pXW6.27; patcGFP::FBF-1) unc-119(ed3) III; him-8(tm611) IV* | *gld-1* prom::patcGFP::FBF-1::*fbf-1 3’UTR* + *unc-119(+)* | UMT392 | this study |
| *fbf-1(ok91) fbf-2(q704) II; mntSi27 (pXW6.26; patcGFP::FBF-2) unc-119(ed3) III* | *gld-1* prom::patcGFP::FBF-2::*fbf-2 3’UTR* + *unc-119(+)* | UMT382 | this study |
| *fbf-1(ok91) fbf-2(q704) II; mntSi32 (pXW6.32; patcGFP::FBF-2vrm) unc-119(ed3) III* | *gld-1* prom::patcGFP::FBF-2(vrm)::*fbf-2* 3’UTR + *unc-119(+)* | UMT445 | this study |
| *fbf-1(ok91) fbf-2(q704) II; mntSi26 (pXW6.25; patcGFP::FBF-1(FBF-2vr3)) unc-119(ed3) III* | *gld-1* prom::patcGFP::FBF-1(FBF-2vr3)::*fbf-1 3’UTR* + *unc-119(+)* | UMT381 | this study |
| *fbf-1(ok91) fbf-2(q704) II; mntSi31 (pXW6.31; patcGFP::FBF-1(FBF-2vr4)) unc-119(ed3) III* | *gld-1* prom::patcGFP::FBF-1(FBF-2vr4)::*fbf-1 3’UTR* + *unc-119(+)* | UMT418 | this study |
| *fbf-1(ok91) II; axIs1471 (pCM4.06; GFP::FBF-1)* | *pie-1* prom::GFP::FBF-1::*fbf-1 3’UTR* + *unc-119(+)* | UMT240 | this study |
| *fbf-1(ok91) II; axIs2000 (pEV1.05; LAP::FBF-2)* | *pie-1* prom::LAP::FBF-2::*fbf-2 3’UTR* + *unc-119(+)* | UMT136 | this study |
| *fbf-1(ok91) II; mntSi26 (pXW6.25; patcGFP::FBF-1(FBF-2vr3)) unc-119(ed3) III* | *gld-1* prom::patcGFP::FBF-1(FBF-2vr3)::*fbf-1 3’UTR* + *unc-119(+)* | UMT402 | this study |
| *fbf-1(ok91) II; mntSi31 (pXW6.31; patcGFP::FBF-1(FBF-2vr4)) unc-119(ed3) III* | *gld-1* prom::patcGFP::FBF-1(FBF-2vr4)::*fbf-1 3’UTR* + *unc-119(+)* | UMT419 | this study |
| *fbf-1(ok91) II; mntSi16 (pXW6.05; LAP::FBF-2(vrm)) unc-119(ed3) III* | *fbf-2* prom::LAP::FBF-2(vrm)::*fbf-2* 3’UTR + *unc-119(+)* | UMT256 | this study |
| *fbf-2(q738) II; axIs1471 (pCM4.06; GFP::FBF-1) IV* | *pie-1* prom::GFP::FBF-1::*fbf-1 3’UTR* + *unc-119(+)* | UMT166 | this study |
| *fbf-2(q738) II; axIs2000 (pEV1.05; LAP::FBF-2)* | *pie-1* prom::LAP::FBF-2::*fbf-2 3’UTR* + *unc-119(+)* | UMT137 | (Wang et al., 2016) |
| *fbf-2(q738) II; mntSi26 (pXW6.25; patcGFP::FBF-1(FBF-2vr3)) unc-119(ed3) III* | *gld-1* prom::patcGFP::FBF-1(FBF-2vr3)::*fbf-1 3’UTR* + *unc-119(+)* | UMT412 | this study |
| *fbf-2(q738) II; mntSi31 (pXW6.31; patcGFP::FBF-1(FBF-2vr4)) unc-119(ed3) III* | *gld-1* prom::patcGFP::FBF-1(FBF-2vr4)::*fbf-1 3’UTR* + *unc-119(+)* | UMT417 | this study |
| *fbf-2(q738) II; mntSi16 (pXW6.05; LAP::FBF-2(vrm)) unc-119(ed3) III* | *fbf-2* prom::LAP::FBF-2(vrm)::*fbf-2* 3’UTR + *unc-119(+)* | UMT297 | this study |
|  | | | |
