# Supplementary material for: Antagonistic control of Caenorhabditis elegans germline stem cell proliferation and differentiation by PUF proteins FBF-1 and FBF-2
Source: eLife. 2020 Aug 17;9:e52788. doi: 10.7554/eLife.52788 (PMC7467723; doi:10.7554/eLife.52788)
Supplement: Supplementary file 2. [file elife-52788-supp2.docx]

**Supplementary Table 2**

Antibodies used in the study

| **Antibody** | **Source or Reference** | **Catalog Number or Designation** | **Dilution** |
| --- | --- | --- | --- |
| Immunostaining, Primary Antibodies | | | |
| Mouse anti-FLAG, IgG1 | Sigma-Aldrich | F1804 | 1:1,000 |
| Rabbit monoclonal anti-GFP, IgG | Thermo-Fisher | G10362 | 1:200 |
| Mouse anti-phospho-Histone H3 pSer10 6G3, IgG1 | Cell Signaling Technology | 9706 | 1:400 |
| Rabbit anti-REC-8, IgG | Novus Biologicals | 29470002 | 1:500 |
| Mouse anti-PGL-1, IgM | DSHB | K76 | 5.2 μg/ml |
| Rabbit anti-FBF-1, IgG | (Voronina et al., 2012) | PA2388 | 3.5 μg/ml |
| Immunostaining, Secondary Antibodies | | | |
| Alexa Fluor 594-conjugated goat anti-mouse IgG (H+L) | Jackson ImmunoResearch |  | 1:500 |
| Alexa Fluor 594-conjugated goat anti-rabbit IgG (H+L) | Jackson ImmunoResearch |  | 1:500 |
| Alexa Fluor 488-conjugated goat anti-rabbit IgG | Jackson ImmunoResearch |  | 1:500 |
| Alexa-594 goat anti-mouse IgM | Jackson ImmunoResearch |  | 1:500 |
| PLA, Primary Antibodies | | | |
| Mouse anti-FLAG, IgG1 | Sigma-Aldrich | F1804 | 1:1,000 |
| Rabbit monoclonal anti-GFP, IgG | Thermo-Fisher | G10362 | 1:40,000 |
| Western blotting, Primary Antibodies | | | |
| Mouse anti-FLAG, IgG1 | Sigma-Aldrich | F1804 | 1:1,000 |
| Mouse anti-Tubulin DM1A | Sigma-Aldrich | T6199 | 1:300 |
| Rabbit anti-FBF-1, IgG | (Voronina et al., 2012) | PA2388 | 5.2 μg/ml |
| Western blotting, Secondary Antibodies | | | |
| HRP anti-mouse | Jackson ImmunoResearch |  | 1:5000 |
| HRP anti-rabbit | Jackson ImmunoResearch |  | 1:5000 |
