# Supplementary material for: Antagonistic control of Caenorhabditis elegans germline stem cell proliferation and differentiation by PUF proteins FBF-1 and FBF-2
Source: eLife. 2020 Aug 17;9:e52788. doi: 10.7554/eLife.52788 (PMC7467723; doi:10.7554/eLife.52788)
Supplement: Supplementary file 3. — Percent embryos failing to hatch was determined 24 hr after eggs were deposited on a plate. The data were obtained in two independent experiments. [file elife-52788-supp3.docx]

**Supplementary Table 3**

Embryo lethality resulting from CCR4-NOT knockdown in the parent generation.
Percent embryos failing to hatch was determined 24 hours after eggs were deposited on a plate. The data were obtained in two independent experiments.

| genotype | RNAi | *N* | % Embryo lethality |
| --- | --- | --- | --- |
| wild type | control | 483 | 0 |
|  | *let-711* | 287 | 49 |
|  | *ccf-1* | 166 | 26 |
|  | *ccr-4* | 556 | 19 |
| *fbf-1(lf)* | control | 342 | 1 |
|  | *let-711* | 209 | 62 |
|  | *ccf-1* | 127 | 41 |
|  | *ccr-4* | 271 | 20 |
| *fbf-2(lf)* | control | 361 | 0 |
|  | *let-711* | 375 | 63 |
|  | *ccf-1* | 108 | 77 |
|  | *ccr-4* | 191 | 22 |
